# Prevalence of exhaustion symptoms and associations with school level, length of work experience and gender: a nationwide cross-sectional study of Swedish principals

**DOI:** 10.1186/s12889-021-10317-7

**Published:** 2021-02-10

**Authors:** Roger Persson, Ulf Leo, Inger Arvidsson, Carita Håkansson, Kerstin Nilsson, Kai Österberg

**Affiliations:** 1 Department of Laboratory Medicine, Division of Occupational and Environmental Medicine, Lund University, SE-22185 Lund, Sweden; 2 Department of Psychology, Lund University, SE-22100 Lund, Sweden; 3 Centre for Medicine and Technology for Working Life and Society (Metalund), Lund, Sweden; 4 Centre for Principal Development, Umeå University, Umeå, Sweden; 5 Department of Health Sciences, Kristianstad University, Kristianstad, Sweden

**Keywords:** Exhaustion, KEDS, LUCIE, School leaders, Self-rated health, Stress, Wellbeing, Work

## Abstract

**Background:**

While poor mental health and psychiatric disorders attributed to stressful work conditions are a public health concern in many countries, the health consequences of the occupational stress experienced by school principals is an understudied issue. Although current data is lacking, some research suggests that principals have a stressful work situation that eventually may lead to burnout and exhaustion disorder, thus negatively affecting the ability of principals to function as leaders. To gauge the situation in Sweden, and as a basis for future preventive actions, we examined to what extent principals displayed signs of exhaustion and whether the prevalence rates of exhaustion differed across school levels, length of work experience as a principal, and gender.

**Methods:**

Principals (*N* = 2219; mean age 49 years [SD 7 years]; 78% women) working at least 50% in pre-schools, compulsory schools, upper secondary schools or adult education completed a cross-sectional web survey entailing two validated inventories: The Karolinska Exhaustion Disorder Scale (KEDS) and the Lund University Checklist for Incipient Exhaustion (LUCIE). Data was analysed using traditional non-parametric methods. Gender stratification achieved covariate balance when analysing school level and length of work experience.

**Results:**

Altogether, 29.0% of the principals met the exhaustion criteria in KEDS. The prevalence rates for the four LUCIE-steps of increasing signs of exhaustion were: no signs of stress, 48.8%; weak signs of stress, 25.6%; clear signs of stress but no exhaustion, 15.4%; possible exhaustion disorder, 10.2%. Compared with male principals, female principals reported more signs of possible exhaustion disorder in both LUCIE and KEDS. School level was not associated with reports of exhaustion symptoms in neither LUCIE nor KEDS. Among male principals, length of work experience was associated with exhaustion symptoms in KEDS.

**Conclusions:**

A large group of Swedish principals working in pre-schools, compulsory schools, upper secondary schools or adult education displayed a symptomatology of signs of exhaustion that if sustained might lead to poor health. This observation suggests that education authorities, or other relevant stakeholders, ought to take some form of preventive action. However, effective combinations of individual, group, organisational, and/or societal preventive activities remain to be identified and tested.

## Background

In contrast to research on teachers occupational stress, health and wellbeing (e.g., [1–6]), the health consequences of occupational stress on school principals has received less research attention. The need for new insights in this understudied area of concern have been acknowledged [7–9]. Indeed, as leaders, principals occupy a key position and have extensive responsibilities and accountability in several areas such as the work environment, pedagogical development, staffing and budgets, etc. [10, 11]. So, as part of the educational system, the actions of principals may contribute to public health via their influences on teachers, children and youth.

To function in their work role, principals, just as any employee, need proper organisational preconditions, motivation, and good health [12, 13]. It is therefore conspicuous that studies from, for example, Australia [8, 14–18], Belgium [19], Canada [9], Israel [20, 21], Ireland [7], Malaysia [22], United Kingdom [23] and the United States of America [24, 25] collectively indicate that principals have a stressful work situation that put them at risk for work overload, chronic stress and, eventually, conditions such as burnout [26], exhaustion disorder (ED) [27] or other forms of poor health (e.g., cardiovascular disease) [14, 28]. In addition, overstressed principals may contribute to secondary effects by discontinuing their job or negatively influencing the health and wellbeing of staff and students via sub-optimal leadership (e.g., hasty decision-making, lack of strategic planning, limited access to support, etc.). For example, teachers who report experiencing poor leadership tend to report higher burnout scores [2]. And stress and strain have been reported to bi-directionally transmit between principals and teachers [20].

Nevertheless, Swedish research is lacking, and since many international studies have been made prior to the 2010s, their relevance for understanding contemporary principals’ mental health status is declining. Yet, the workload of principals remains a concern in many countries. And, in 2018, questions on principals’ stress were introduced in the Teaching and Learning International Survey (TALIS) [29]. However, these questions only concern perceived sources of stress and do not gauge the mental health status of principals. Yet, according to TALIS, Swedish principals perceived that bearing too much administrative work, having extra duties due to absent school staff, and accommodating students with special needs to be the three most prevalent sources of stress [30].

The importance of monitoring a broad range of psychosocial hazards at work (e.g., violence, harassment, work-life balance, and work-related stress) has been recognized [31]. And the potential consequences of poorly managed psychosocial hazards such as poor mental health and psychiatric disorders attributed to stressful work conditions is not only a concern for principals. It is also a lasting public health concern in many countries [32–36]. And in response to this concern, the Swedish National Board of Health and Welfare (NBHW) adopted the diagnosis of exhaustion disorder (ED) in 2005 [27, 37]. Incorporated within the diagnostic entity F43 “*Reactions to severe stress and adjustments disorders*” and ICD-10 code F43.8 “*Other reactions to severe stress*” [37], ED was designated F43.8A. In contrast to burnout, which focuses on various psychological manifestations of exhaustion (e.g., exhaustion, cynicism and decreased professional efficacy) [26], ED emphasises the physiological aspects that are associated with the excessive and often paralyzing feelings of tiredness, which is a core symptom in both burnout and ED [38, 39]. In contrast to the ED diagnosis, the burnout diagnosis (Z73.0) carries a lower weight in that it is placed in the ICD-10 category “Problems related to life management difficulty” [37]. Observably, this lower diagnostic weight appears to be retained in the forthcoming ICD-11 [40]. However, in ICD-11, the conceptualisation of burnout (QD85) has been improved and clarified. And it is now explained that burnout is a result from chronic workplace stress and a phenomena that is constrained to the occupational context and distinct from adjustment disorder (6B43) [40].

The ED diagnosis was introduced to facilitate the clinical management of people who had sought help for a work-related symptomatology characterised by psychological and physiological complaints relating to excessive tiredness without co-occurring signs of psychiatric morbidity or other conditions (e.g., depression, diabetes, general anxiety, heart disease, substance abuse, thyroid disease, etc.). Presumably, ED is the result of insufficient rest and recovery from very frequent, or excessively long-lasting, stress responses [27, 41–43]. From this perspective, it should be noted that about 70% of the principals in Sweden are women [44]. And it is known that men and women who perform similar work display similar physiological stress responses [45] and tend to develop similar types of health issues [46]. Accordingly, male and female principals who perform similar work should develop similar health problems. However, on average, women are also known to report more numerous, intense, and frequent bodily symptoms than men [47], and to be more long-term sick-listed than men. This includes sick-listing for psychiatric diagnoses (e.g., depression, anxiety and/or ED), which, since 2014, have been the most common cause of sick-listing in Sweden [48, 49].

Because there are no physiological changes that indisputably define ED, diagnosis and treatment efforts are dependent on analysing contextual circumstances and subjective symptoms. So, to facilitate the detection, monitoring and treatment of ED, the Self-reported Exhaustion Disorder Questionnaire [50] and the Karolinska Exhaustion Disorder Scale (KEDS) [51] were developed. In addition, the Lund University Checklist for Incipient Exhaustion (LUCIE) [52, 53] was developed to assess the earliest signs of exhaustion. Despite conceptual differences, instruments designed to assess symptoms of burnout and ED are known to be positively correlated and thus share common ground [54].

### Objectives

Overstressed principals may develop poor health and contribute to secondary effects by discontinuing their job or negatively influencing the health and wellbeing of staff and students via sub-optimal leadership. Consequently, knowledge about principals’ health status is important when it comes to the planning and prioritising of any action that aim to protect principals’ health and/or to avoid unwanted secondary effects. In the absence of current Swedish and internationally comparable data on principals’ mental health status, the present study aimed to gauge the occurrence of early and manifest signs of exhaustion among Swedish principals. Accordingly, the first aim was to examine to what extent principals reported early signs of exhaustion as well as manifest signs of exhaustion in two validated exhaustion inventories (i.e., LUCIE and KEDS). Secondly, the aim was to examine whether the prevalence rates of exhaustion symptoms differed across various school levels (i.e., pre-school, compulsory school, upper secondary school and adult education), length of work experience as a principal, and gender. Since signs of exhaustion can be assumed to be sensitive indicators of emerging health problems, knowledge of their occurrence and distribution provides a needed basis for decisions on future preventive actions aimed to improve principals’ mental health.

## Methods

### Study design

The present cross-sectional web survey is part of an explorative longitudinal study with two assessments. Using a non-probability purposive sampling strategy, the study targeted Swedish principals at all school levels working at least 50%. Because there is no accessible official register of principals, participants were recruited via a list of e-mail addresses that covered principals who during the period 2008–2017 had participated in training programmes funded and arranged by the Swedish National Agency for Education and run by different universities in Sweden. The survey contained both established questionnaires and scales, as well as project-specific questions and items designed to capture the unique features of the work of principals. The current data were collected during the first assessment with the software Textalk Websurvey (www.textalk.se; Gothenburg, Sweden) between September 25 and October 23, 2018. Four reminders were sent in the month of October. The period was selected with regard to the research questions and to allow assessment during a period with a relatively stable workload in a work year that comprises both peak loads and holidays.

### Participants

**Fig. 1:**
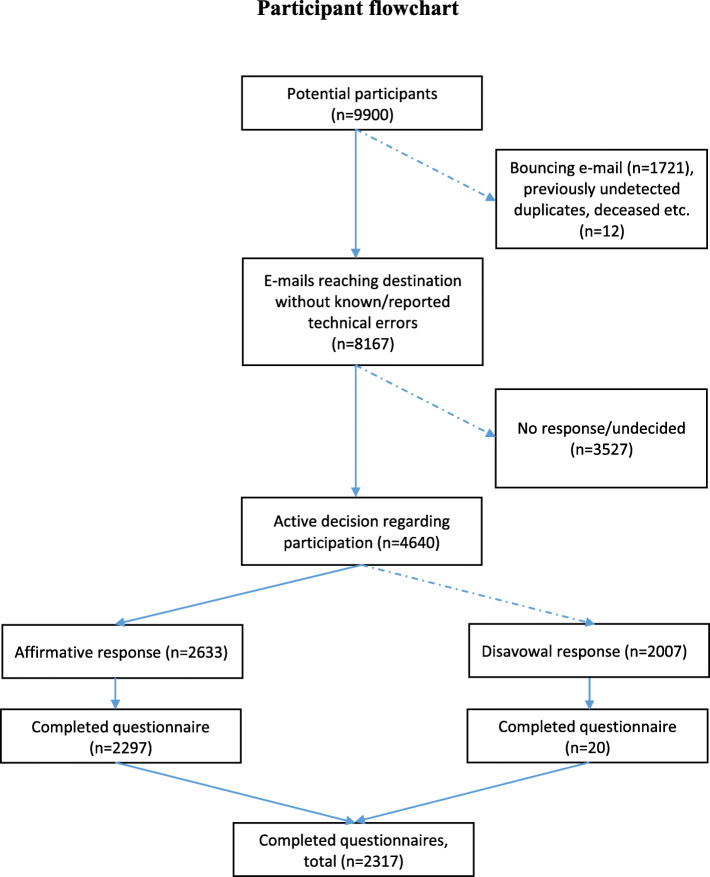
Participant flowchart: Identification of the crude study sample. The figure depicts the participant flow from the first e-mail invitations to the completion of the web-administered questionnaires

With a target of obtaining at least 2000 participants (see Statistical Analysis), 9900 presumptive principals were invited to participate by e-mail. Of these, 4640 potential responders either accepted (*n* = 2633) or declined (*n* = 2007) participation. Ultimately, 2317 individuals completed the entire questionnaire (Fig. 1). In the present analysis, only participants working at least 50% in pre-schools, compulsory schools, upper secondary schools or adult education were included (*N* = 2219). Their mean age was 49.3 years (SD 7.4 years); 77.7% were females (*n* = 1724), 22.1% were males (*n* = 491), and 0.2% (n = 4) did not disclose their gender. The majority of the participants (96.0%) reported working ≥90%, and 18.5% reported that during the preceding 12 months they had surpassed their scheduled work time on a daily basis, whereas 56% had done so a couple of days a week. The most common type of employer comprised municipalities (77.3%); less common were private share holding companies (11.0%) and other employers (11.7%). Table 1 presents additional participant data regarding access to social support in private life, co-habitation status, school level, perceived stress and pressure not attributed to work, geographical origin and length of work experience as a principal. Table 2 present data on the participants’ lifestyle behaviours concerning alcohol intoxication, smoking, and leisure time physical activity levels as assessed by the Saltin-Grimby scale [55, 56].

**Table 1:** Demographic characteristics. Distribution of mean age scores and associated standard deviations (SD), and the proportion of men and women across categories (*N* = 2219)

	Age (years)		Total (*N* = 2219)		^c^Women (*n* = 1724)		^c^Men (*n* = 491)		Chi-square test
	Mean	SD	%	N	%	N	%	N	*P*-value
^d^Access to social support if needed									< 0.001
Yes, a lot	49.5	7.3	43.4	962	46.3_a_	798	33.0_b_	162	
Yes, quite a bit	49.5	7.5	34.5	766	34.1_a_	588	36.0_a_	177	
Yes, to some extent	48.4	7.4	16.5	367	15.0_a_	259	21.8_b_	107	
No	49.7	7.1	5.6	124	4.6_a_	79	9.2_b_	45	
Co-habitant status									0.015
Yes	49.2	7.3	84.9	1885	83.8_a_	1444	89.0_b_	437	
Living apart	50.1	8.0	11.3	251	12.3_a_	212	7.9_b_	39	
No	49.7	7.7	3.7	83	3.9_a_	68	3.1_a_	15	
Job title									0.098
^e^Principal	49.7	7.3	77.8	1726	78.2	1348	76.4	375	
Assistant principal	47.6	7.6	18.0	399	17.2	297	20.6	101	
^f^Other	49.4	7.9	4.2	94	4.6	79	3.1	15	
School level									< 0.001
Pre-school	49.9	7.2	27.9	619	34.7_a_	598	4.1_b_	20	
Pre-school and compulsory school	50.5	7.7	5.0	111	5.4_a_	93	3.7_a_	18	
Compulsory school	49.0	7.2	44.4	986	41.6_a_	718	54.2_b_	266	
Upper secondary school	48.6	7.7	15.4	342	12.1_a_	209	27.1_b_	133	
Adult education	49.5	8.4	7.3	161	6.1_a_	106	11.0_b_	54	
^g^Stress/pressure reported to be unrelated to work									0.874
Yes, a lot	49.3	7.9	9.2	204	9.3	161	8.8	43	
Yes, quite a bit	49.4	7.5	15.7	349	15.5	267	16.5	81	
Yes, to some extent	48.8	7.1	34.5	765	34.2	590	35.2	173	
No	49.7	7.4	40.6	901	41.0	706	39.5	194	
^h^Region of origin									0.051
Stockholm	49.9	7.6	20.0	444	21.1	364	16.3	80	
Västra Götaland	48.5	7.5	14.1	313	13.5	232	16.1	79	
Skåne	49.2	7.4	17.2	381	17.5	301	16.1	79	
Sweden (other)	49.3	7.3	48.7	1080	47.9	826	51.5	253	
^i^Years of work experience as a principal									< 0.001
0 < 3 years	45.6	7.1	19.5	432	20.7_a_	357	14.7_b_	72	
3 < 5 years	47.0	6.5	22.5	500	23.3_a_	402	20.0_a_	98	
5 to 10 years	49.2	6.7	33.9	753	33.9_a_	585	34.0_a_	167	
> 10 years	54.7	6.3	24.1	534	22.0_a_	380	31.4_b_	154	

a and b values in the same row and subtable not sharing the same subscript (a or b) are significantly different at *p* < 0.05 in the two-tailed test of equality for column proportions (Bonferroni adjusted)

^c^Four individuals did not disclose their gender

^d^The question read - “Do you have access to social support if you need it in your private life?”

^e^This category includes the job titles pre-school director (*n* = 556) as well as principal and pre-school director (*n* = 68), which from July 1st 2019 has been retitled to principal

^f^The designation ‘other’ refers to school leaders whose formal title was not ‘Principal’ or ‘Assistant Principal’, who, however, described a combination of responsibilities and duties that was judged to conform with the formal titles of ‘Principal’ or ‘Assistant Principal

^g^The question read - “Have you during the last year experienced stress and/or pressure caused by factors that are unrelated to your work?”

^h^One individual disclosed no region

^i^The outermost categories (i.e., < 1 year, and > 20 years) have been merged with adjacent categories

**Table 2:** Lifestyle behaviors. Distribution of mean age and associated standard deviations (SD), and the proportion of men and women across categories (*N* = 2219)

	Age (years)		Total (*N* = 2219)		^c^Women (*n* = 1724)		^c^Men (*n* = 491)		Chi-square test
	Mean	SD	%	N	%	N	%	N	*P*-value
^d^Alcohol intoxication									< 0.001
Daily or almost daily, A few times a week, Once a week	49.5	8.3	2.6	57	1.5_a_	26	6.3_b_	31	
2–3 times a month, Once a month	48.1	7.0	9.8	217	6.9_a_	119	20.0_b_	98	
A few times every 6-months	47.3	7.1	23.5	521	20.8_a_	359	33.0_b_	162	
More seldom or never	50.2	7.3	64.2	1424	70.8_a_	1220	40.7_b_	200	
^e^Physical activity									< 0.001
Inactive	49.1	7.5	11.6	258	13.0_a_	224	6.7_b_	33	
Light	50.1	7.6	36.9	818	37.9_a_	653	33.6_a_	165	
Medium	49.3	7.0	42.1	934	41.6_a_	717	44.0_a_	216	
Heavy	46.4	7.4	9.4	209	7.5_a_	130	15.7_b_	77	
Smoking									0.083
Yes, daily	49.4	8.9	2.5	55	2.8	49	1.2	6	
Yes, occasionally	49.5	7.7	3.5	77	3.7	63	2.9	14	
No	49.3	7.3	94.1	2087	93.5	1612	95.9	471	
^f^Wish to reduce alcohol consumption									0.001
Yes, and I think I can manage it by my self	50.7	7.3	7.5	167	6.4_a_	110	11.6_b_	57	
Yes, but I need professional support	55.6	2.1	0.2	5	0.2_a_	3	0.4_a_	2	
No	49.2	7.4	83.3	1848	84.2_a_	1452	79.8_b_	392	
Not applicable, I never use alcohol.	49.0	7.3	9.0	199	9.2_a_	159	8.1_a_	40	

a and b = values in the same row and subtable not sharing the same subscript (a or b) are significantly different at *p* < 0.05 in the two-tailed test of equality for column proportions (Bonferroni adjusted)

^c^Four individuals did not disclose their gender;

^d^The question read - “How often, during the last 12-months, have you consumed alcohol to the extent that you have been intoxicated (drunk)? No test of equality for column proportions for the first category “Daily almost daily” due to too few participants

^e^ Inactive = almost completely inactive (e.g., primarily reading, watching television, watching movies, using computers or doing other sedentary activities). Light = light physical activity for at least 4 h/week (e.g., riding a bicycle or walking to work, walking with the family, gardening, fishing, table tennis, bowling etc.). Medium = performing regular physical activity and training for at least 2–3 h/week (e.g., spending time doing heavy gardening, running, swimming, playing tennis, badminton, calisthenics and similar activities). Heavy = engaging in regular hard physical training for competitive sports several times per week (e.g. spending time running, orienteering, skiing, swimming, playing football, handball etc.)

^f^The question read - “Do you wish to reduce your alcohol consumption”

### Measures

Background information was collected on *age* (i.e., year of birth), *co-habitation status* (i.e., yes, no, living apart), *job title* (i.e., principal, assistant principal, other), *lifestyle behaviours* (i.e., alcohol intoxication, smoking and leisure-time physical activity levels [the Saltin-Grimby Physical Activity Level Scale] [55, 56]; see Table 2 for response alternatives), *perceived access to social support in private life if needed* (i.e., yes, a lot; yes, quite a bit; yes, to some extent; no)*, perceived stress and pressure during the last year that is caused by non-work related factors* (i.e., yes, a lot; yes, quite a bit; yes, to some extent; no)*.*

*Length of work experience as a principal* (i.e., < 1 year, 1 to 3 years, > 3 to 5 years, > 5 to 10 years, > 10 to 20 years, > 20 years), *School level* (i.e., pre-school, pre-school and compulsory school, compulsory school, upper secondary school, and adult education), and *gender* (i.e., female, male, and other/do not want to disclose) were used to define subgroups of principals.

*The Karolinska Exhaustion Disorder Scale* (KEDS) comprises nine items that refer to the past 2 weeks and which cover the following domains: (1) ability to concentrate, (2) memory, (3) physical stamina, (4) mental stamina, (5) recovery, (6) sleep, (7) hypersensitivity to sensory impressions, (8) experience of demands, and (9) irritation and anger [51]. Each item is responded to on a 7-point scale (0–6). Short descriptive verbal phrases serve as anchors for the scale steps 0, 2, 4 and 6, but not for 1, 3 and 5. Higher values reflect more severe symptoms, and the sum score (0 to 54) is used as an outcome, with a score ≥ 19 indicating possible ED [51, 57].

*The Lund University Checklist for Incipient Exhaustion* (LUCIE) comprises 28 items (statements) that refer to the past 4 weeks and which cover the following six domains: (a) sleep and recovery (3 items), (b) separation between work and spare time (4 items), (c) sense of community and support in the workplace (2 items), (d) managing work duties and personal capabilities (5 items), (e) private life and spare time activities (3 items), and (f) health complaints (11 items) [52, 53, 58]. The items are responded to on a 4-point scale: 1 = not at all, 2 = somewhat, 3 = quite a bit, and 4 = very much. For a detailed description of the scoring, see Persson et al. [53]. In brief, the scoring builds on two supplementary algorithms comprising two separate but supplementary scales: the *Stress Warning Scale* (SWS) and the *Exhaustion Warning Scale* (EWS). Specifically, the *SWS* is sensitive to milder signs of incipient exhaustion, whereas the *EWS* reflects more severe signs of exhaustion*.* The main difference between the two scales concerns the *intensity* of the replies. The EWS score is primarily based on replies at the highest level of the 4-point response scale (i.e., ‘very much’). In contrast, the SWS score also includes replies on the adjacent lower level (i.e., ‘quite a bit’). In practice, these two scales are combined into a four-step ranking of incremental stress symptomatology, with the highest level possibly being indicative of ED: Step 1-GG (SWS green zone and EWS green zone), indicating no signs of stress; Step 2-YG (SWS yellow zone and EWS green zone), indicating weak signs of stress; Step 3-RG (SWS red zone and EWS green zone), indicating clear signs of stress but not of exhaustion; and Step 4-RR (SWS red zone and EWS red zone), indicating severe stress signs and possible exhaustion disorder.

The reason for using both KEDS and LUCIE was that both inventories were considered contextually relevant in that they gauge common manifestations of prolonged stress and often are used in parallel in clinical practice [59, 60]. However, the differences in intended purpose, design, layout, scoring procedures and the period the questions refer to makes LUCIE and KEDS supplementary rather than interchangeable.

### Statistical analysis

The quality of in-data was ensured by: (a) Removing duplicates from the e-mail list prior to inviting the principals (the nationwide reach of the e-mail list was ascertained beforehand). (b) Applying a one-month response window, up to four reminders and allowing interim savings of partially filled in questionnaires. (c) Setting up the questionnaire to minimize the occurrence of internal missing values. (d) Using validated questionnaires to provide common definitions of central concepts as well as project-specific items, designed to capture specific aspects of the principals background not covered by other measures.

The IBM SPSS software (version 26.0.0.1) was used for statistical analysis. Two-tailed *p*-values ≤0.05 were considered statistically significant. The target level of 2000 participants was based on the knowledge that circa 10 and 5% in a highly educated part of the general population can be expected to display signs of exhaustion in KEDS and LUCIE, respectively [53]. Hence, this level was judged to give sufficient statistical power and to allow relevant subgroup comparisons. Because LUCIE scores are on a classificatory scale (or on an ordinal scale of increasing frequency of signs of exhaustion), and because comparisons had to be made across groups of different sizes, the data were analysed with traditional methods for non-parametric statistics. Confidence intervals (95%) for prevalence rates were constructed with a bootstrap procedure. For nominal or categorical data, between-group comparisons were made using Pearson’s independent chi-square test and pairwise follow-up tests with a Bonferroni-adjusted z-test for column proportions.

Preliminary analyses indicated that the level of exhaustion signs in both KEDS and LUCIE did not differ across job title (i.e., principal, assistant principal, and “other” titles signifying equivalent responsibilities as being a principal or assistant principal). The participants were therefore treated as one group (i.e., principals) in the statistical analyses. Preliminary analyses also identified an uneven distribution of men and women across the categories of length of work experience and school level. To avoid bias due to gender, gender-stratified analyses were applied when appropriate. Age scores were typically unrelated to the other variables, except for a positive correlation with length of work experience. Because this dependency was expected, and presumably part of the causal chain, we did not conduct age-stratified analyses. However, due to the small number of responses in the most extreme categories on the work experience item, the outermost response categories were merged with adjacent categories. This created a four-step ordinal scale of increasing years of work experience as a principal: 0 to 3 years, > 3 to 5 years, > 5 to 10 years, and > 10 years.

## Results

The prevalence rates for the four incremental LUCIE-steps of exhaustion signs (from no exhaustion to possible exhaustion disorder) were: Step 1-GG = 48.8% (*n* = 1083; 95% CI = 46.7 to 50.9%); Step 2-YG = 25.6% (*n* = 569; 95% CI = 23.9 to 27.5%); Step 3-RG = 15.4% (*n* = 341; 95% CI = 13.9 to 16.9%); and Step 4-RR = 10.2% (*n* = 226; 95% CI = 9.0 to 11.5%). Compared to male principals, female principals had higher prevalence rates of exhaustion in Step 4-RR and lower prevalence rates in Step 1-GG. There was no difference between men or women with regard to the intermediate steps Step 2-YG and Step 3-RG (Table 3).

**Table 3:** Distribution of mean age and associated standard deviations (SD), and proportion of female and male principals and assistant principals across KEDS and LUCIE categories (*N* = 2219)

	Age (years)		^c^Women (*n* = 1724)		^c^Men (*n* = 491)		Chi-square test
	Mean	SD	%	N	%	N	*P*-value
KEDS (0–54)							0.003
Normal (< 19)	49.5	7.3	69.4_a_	1196	76.4_b_	375	
Exhaustion (≥ 19)	48.8	7.6	30.6_a_	528	23.6_b_	116	
LUCIE							0.003
Step 1-GG	49.2	7.5	47.3_a_	815	53.8_b_	264	
Step 2-YG	49.7	7.0	26.2_a_	452	23.8_a_	117	
Step 3-RG	48.9	7.8	15.2_a_	262	16.1_a_	79	
^d^Step 4-RR	49.4	7.5	11.3_a_	195	6.3_b_	31	

a and b = values in the same row and subtable not sharing the same subscript (a or b) are significantly different at *p* < 0.05 in the two-tailed test of equality for column proportions (Bonferroni adjusted)

^c^Four individuals did not disclose their gender

^d^The rare LUCIE combination of SWS yellow + UWS red (*n* = 10) are included in this category

In total, 29.0% (*n* = 644; 95% CI = 27.2 to 30.9%) of the principals met the exhaustion criteria in KEDS. More female principals reported signs of exhaustion than their male colleagues (Table 3). The mean KEDS sum score in the total study sample was 14.4 (SD = 8.4; 95% CI = 14.0 to 14.8). For principals who met the exhaustion criteria in KEDS, the mean KEDS sum score was 24.9 (SD = 5.3; 95% CI = 24.5 to 25.3), whereas principals without an exhaustion warning had a mean KEDS sum score of 10.2 (SD = 4.9; 95% CI = 9.9 to 10.4).

### Associations with school level

Chi-square tests indicated no difference in the prevalence rates of exhaustion in LUCIE or KEDS that was contingent on school level (Table 4). Similarly, the gender stratified analyses disclosed no differences between school levels and female and male principals’ reports of exhaustion in LUCIE (*p* = 0.874 and 0.336, respectively) or KEDS (*p* = 0.434 and 0.239, respectively) (data not shown).

**Table 4:** Distribution of KEDS and LUCIE scores across school levels (*N* = 2219)

	Pre-School (*n* = 619)		Pre and compulsory school (*n* = 111)		Compulsory school (*n* = 986)		High school (*n* = 342)		Adult education (*n* = 161)		Chi-square test
	%	N	%	N	%	N	%	N	%	N	*P*-value
KEDS (0–54)											0.481
Normal (< 19)	70.1	434	73.0	81	70.6	696	74.6	255	67.7	109	
Exhaustion (≥ 19)	29.9	185	27.0	30	29.4	290	25.4	87	32.3	52	
LUCIE											0.178
Step 1-GG	52.3	324	44.1	49	46.5	458	50.6	173	49.1	79	
Step 2-YG	24.4	151	21.6	24	26.9	265	27.2	93	22.4	36	
Step 3-RG	13.1	81	20.7	23	17.0	168	12.3	42	16.8	27	
^a^Step 4-RR	10.2	63	13.5	15	9.6	95	9.9	34	11.8	19	

^a^The rare LUCIE combination of SWS yellow + UWS red (*n* = 10) are included in this category

### Associations with length of work experience as a principal

In the total study sample, a chi square test indicted no difference in prevalence rates across LUCIE categories contingent on the length of work experience as a principal (*p* = 0.098). In contrast, a chi-square test indicated that the prevalence rates of exhaustion in KEDS was contingent on the length of work experience as a principal (*p* = 0.014) (Table 5). Yet, the Bonferroni adjusted post-hoc tests did not reach statistical significance even when the pattern of prevalence rates of exhaustion seemed to decline with increasing length of work experience. A gender-stratified analysis showed that the prevalence rate for exhaustion in KEDS for male principals with length of work experience from 3 to 5 years (37.8%) was higher than the rates observed among their male colleagues with 5 to 10 years of work experience (21.0%), or with more than 10 years of work experience (16.9%) (χ2 (3) =15.457, *p* = 0.001) (data not shown). No differences were observed among women (KEDS *p* = 0.298 and LUCIE *p* = 0.089) (data not shown).

**Table 5:** Distribution KEDS and LUCIE scores across length of work experience as a principal (*N* = 2219)

	0 to 3 years (*n* = 432)		> 3 to 5 years (*n* = 500)		> 5 to 10 years (*n* = 732)		> 10 years (*n* = 534)		Chi-square test
	%	N	%	N	%	N	%	N	*P*-value
KEDS (0–54)									0.014
Normal (< 19)	67.1_a_	290	67.6_a_	338	72.8_a_	548	74.7_a_	399	
Exhaustion (≥ 19)	32.9_a_	142	32.4_a_	162	27.2_a_	205	25.3_a_	135	
LUCIE									0.093
Step 1-GG	44.4	192	47.0	235	49.7	374	52.8	282	
Step 2-YG	26.2	113	24.8	124	26.2	197	25.3	135	
Step 3-RG	18.3	79	17.4	87	15.1_._	114	11.4	61	
^a^Step 4-RR	11.1	48	10.8	54	9.0	68	10.5	56	

a = values in the same row and subtable not sharing the same subscript (a or b) are significantly different at *p* < 0.05 in the two-tailed test of equality for column proportions (Bonferroni adjusted)

^a^The rare LUCIE combination of SWS yellow + UWS red (*n* = 10) are included in this category

## Discussion

The present study aimed to gauge the occurrence of early and manifest signs of exhaustion among Swedish principals. The results showed that 29% of the principals met the exhaustion criteria in KEDS (i.e., a score ≥ 19), whereas 25% had stress (Step 3-RG =15%) or exhaustion (Step 4-RR = 10%) warnings in LUCIE. These prevalence rates are noticeably higher than the prevalence rates observed in a Swedish study sample drawn from a highly educated part of the general population. In this population sample, the occurrence of exhaustion was 5% in LUCIE and 13% in KEDS [53, 54]. The prevalence rates of stress and exhaustion warnings in LUCIE are also higher when compared with data from another Swedish study comprising 863 leaders and employees in a politically governed regional organisation [61]. In this organisation sample, the occurrence of stress and exhaustion warnings in LUCIE were 8 and 5.5%, respectively [61]. In addition, the prevalence rates of signs of exhaustion in LUCIE were also higher among the principals when compared with a Swedish occupational sample consisting of 3658 occupational therapists (94% females) [62]. In this sample of occupational therapists, the occurrence of stress and exhaustion warnings in LUCIE were 11 and 7%, respectively.

Furthermore, a comparison between the group of principals who met the exhaustion criteria in KEDS (i.e., a score ≥ 19) and 698 care-seeking patients with diverse occupational backgrounds [57] also reveal that the principals expressed a noticeable level of exhaustion complaints. While the principals had a lower mean KEDS sum score than the care-seeking patients who received “stress-related diagnoses (i.e., F43)”, they scored almost on par with the care-seeking patients who received “psychiatric diagnoses other than stress (i.e., F [except F43] + Z56)” and clearly higher than care-seeking patients who received “somatic diagnoses (other codes than F-codes and Z56)” [57]. Moreover, when comparing the principals who met the exhaustion criteria in KEDS and a group of 390 Swedish patients undergoing a 24-week multimodal treatment for their diagnosed and pronounced manifestations of stress-related exhaustion [63], the principals scored substantially lower on KEDS than the patient group did immediately before their treatment. However, compared with the patients post assessment and follow-up scores 12 month later, the principals who met the exhaustion criteria in KEDS scored at a similar level as the patient group on both occasions at which the patients’ average scores remained above the level for exhaustion indication in KEDS [63].

Altogether, the results suggest that a large group of principals have reduced mental health. This observation aligns with the few previous studies that have contained validated and specific scales for mental health [8, 20, 23]. Comparison across studies should, however, be made with caution due to differences in study sample compositions, the instruments used, the year data was collected, and variations across countries that are contingent on variations in the educational system. In addition, due to the differences in questionnaire design, layout, scoring procedures and the period the questions refer to; caution should be exercised when comparing LUCIE and KEDS scores. For example, the 28-item LUCIE covers the spectra from the earliest signs of exhaustion to heavy signs possibly indicative of ED by asking for the occurrence and severity of various symptoms in six domains the last 4 weeks [53]. In contrast, the 9-item KEDS focus on assessing symptoms of exhaustion disorder and comprises nine domains during the last 2 weeks [51]. And in a previous study, we observed a low agreement between LUCIE and KEDS classifications of exhaustion [53]. On the other hand, LUCIE and KEDS scores are positively correlated to each other and to the Shirom-Melamed Burnout Questionnaire [64], which suggest that these instruments share common ground [53, 54].

Nevertheless, female principals reported more signs of exhaustion than their male colleagues in both LUCIE (11% vs. 6%, respectively) and KEDS (31% vs. 24%, respectively). This aligns with the fact that women compared with men tend to report more intense, numerous and frequent bodily symptoms [47], and have higher prevalence rates of symptoms of depression and depression diagnoses, in which exhaustion is a core symptom [65]. Observably, many explanations have been proposed as contributing factors. Examples are biological differences, variations in symptom appraisal and symptom perception, socialisation and social roles, abuse and trauma, and gender bias in research etc. [47, 66]. Nonetheless, that female principals report more exhaustion symptoms than male principals align with previous studies in which gender differences in both LUCIE [52] and KEDS [53] have been reported. However, gender differences have not always consistently been observed in LUCIE [53] or KEDS [57]. It is unclear why female principals in the present study report more exhaustion symptoms than their male colleagues. In light of the background questions, a differential exposure to non-work related stress, a lack of access to social support in the private life, or lifestyle differences (i.e., frequency of alcohol intoxication, smoking, and leisure-time physical activity) appear as unlikely explanations for this gender difference. Indeed, female and male principals reported similar levels of exposure to non-work-related stress. And while female principals reported cohabiting to a slightly lesser extent, they did report a greater access to social support in their private life. While female principals reported being less physically active during their leisure time than male principals, they reported similar levels of smoking and being intoxicated by alcohol more seldomly than their male counterparts. The significance of any gender difference in lifestyle behaviours is, however, obscured by the fact that the principals’ lifestyle habits in general suggest that they are ostensibly as healthy as, or healthier, than the general population in Sweden [67]. For example, that only 5% of the principals reported being daily or occasional smokers, and that 12% reported having sedentary leisure time, are more favourable than the national estimates of 14% (2018) and 16% (2015) for the population in the ages 45 to 64, respectively [67]. That fewer principals smoke than in the general population agrees with their relatively high socioeconomic status [68] and with a previous observation in an Australian study [14].

Interestingly, no differences were found in relation to school level. This contrast with previous observations from United Kingdom in a male-dominated study sample [23]. In this large-scale study, the level of mental ill health (as assessed by the Crown Crisp Experiential Index) was inversely related to school level. Accordingly, the highest level of mental ill health was found in primary schools followed by secondary schools and further/higher education institutions [23]. More recent studies have either kept school level constant [7] or have not included school level as a factor in the statistical analyses [8]. However, for the reasons previously outlined above, comparison across studies should be made with caution.

Although a chi-square test initially indicated an association between KEDS and length of work experience as a principal, post-hoc testing failed to confirm any difference. Nevertheless, a gender-stratified analysis indicated that male principals with 3 to 5 years of work experience had higher prevalence rates of exhaustion signs in KEDS when compared with their male colleagues with 5 to 10 years of work experience, or with more than 10 years of work experience. However, given that length of work experience as a principal was correlated with the participants’ chronological age, it is plausible that “third-variable” factors confound the analysis. For example, in an Australian study, it was observed that older principals reported lower levels of stress and burnout than younger principals, whereas work experience did little to protect against the reporting of adverse psychosocial factors [8]. Anyhow, the absence of clear associations with school level and length of work experience may indicate that these two higher-order constructs either have a low explanatory power for principals’ mental health or that they are just too crude as analytical units.

### Strengths and limitations of the study

That the participating principals worked in different types of schools, had varying degrees of work experience, and entailed responders from all 21 counties (and 277 of the 290 municipalities) in Sweden increases the ecological validity of the results. Similarly, the use of two validated exhaustion inventories increases the accessibility of the results. And the large sample size contributes high power to the statistical analyses and thus provides good possibilities for detecting relevant associations. However, since it was not possible to use a randomized selection procedure that considered regional variations, we had to recruit the participants via a non-random procedure. Accordingly, the representativeness of subunits (e.g. counties or municipalities) could not be ensured. Therefore, we did not consider it meaningful to weigh the estimates, or in other ways control, for geographical origin. In addition, since the study was carried out prior to the pandemic outbreak of the Coronavirus disease-19, the results reflect the pre-pandemic situation.

Another concern is the low response rate. Only 26% (*n* = 2317) of all 9900 invited individuals completed the questionnaire. However, the low response rate is to a large extent likely due to over-coverage. Indeed, since the e-mail list we invited from was a compiled census covering the years 2008 to 2017, and that there is a high job turnover among Swedish principals [69], it is likely that we have invited participants who no longer represented the target population. Nevertheless, of the 4640 principals who we did reach, about 50% completed the full survey. Although a higher response rate would have been desirable, the response rate among the principals that we did reach is similar to the response rate in other large-scale surveys targeting the general population [70], police employees [71], and head teachers and principals [23], and enhance the credibility of the results.

Finally, the large number of statistical comparisons makes it advisable to view single significance tests with caution. Given the aim of the present study, and as a reasonable way to balance the risk of committing type I and type II errors, the 0.05 level was selected throughout in combination with exact *p*-values and Bonferroni adjusted post-hoc tests.

## Conclusion

Close to one third of the principals met the KEDS exhaustion criteria, and on LUCIE every fourth principal showed severe stress signs or possible exhaustion disorder. And a larger proportion of women than men met the exhaustion criteria in KEDS and reported possible exhaustion disorder in LUCIE. Thus, a large group of Swedish principals working in pre-schools, compulsory schools, upper secondary schools or adult education displayed a symptomatology of signs of exhaustion that, if sustained, might lead to poor health. The scope and seriousness of complaints suggest that education authorities, or other relevant stakeholders, ought to take some form of preventive actions. However, effective combinations of individual, group, organisational and/or societal preventive activities remain to be identified and tested.

## Data Availability

Consistent with the study protocol approved by the Regional Ethical Review Board, anonymised data is stored locally at the Division of Occupational and Environmental Medicine, Lund University, Lund, Sweden. In accordance with the ethical approval, crude data is not to be published on the internet. Access to data will be granted to eligible researchers wanting to audit our research. Requests should be directed to the corresponding author.

## Abbreviations

CI: Confidence Interval
ED: Exhaustion Disorder
EWS: Exhaustion Warning Scale
ICD-10: International Statistical Classification of Diseases and Related Health Problems 10th Revision
KEDS: Karolinska Exhaustion Disorder scale
LUCIE: Lund University Checklist for Incipient Exhaustion
M: Mean
*P*-value: Probability value
SD: Standard Deviation
Step 1-GG: LUCIE SWS Green zone and EWS Green zone
Step 2-YG: LUCIE SWS Yellow zone and EWS Green zone
Step 3-RG: LUCIE SWS Red zone and EWS Green zone
Step 4-RR: LUCIE SWS Red zone and EWS Red zone
SWS: Stress Warning Scale
